# Exploring participant appreciation of group-based principles for action in community-based physical activity programs for socially vulnerable groups in the Netherlands

**DOI:** 10.1186/s12889-015-2515-6

**Published:** 2015-11-25

**Authors:** Marion Herens, Annemarie Wagemakers, Lenneke Vaandrager, Maria Koelen

**Affiliations:** Health and Society, Department of Social Sciences, Wageningen University, Hollandseweg 1, PO Box 8130, 6700 EW Wageningen, The Netherlands

## Abstract

**Background:**

Physical inactivity is a core risk factor for non-communicable diseases. In the Netherlands, socially vulnerable groups are relatively less active than groups with higher socio-economic status. Community-based health-enhancing physical activity (CBHEPA) programs aim to empower socially vulnerable groups by improving participants’ health and wellbeing through physical activity. CBHEPA programs often revolve around group-based principles for action, such as active participation, enjoyment, and fostering group processes. As such principles are rarely made explicit, our study aims to identify which of the group-based principles for action are considered important by participants.

**Methods:**

Respondents (*n* = 76) from ten focus groups scored their individual appreciation of group-based principles for action – active participation, enjoyment, and fostering group processes – on a three-point, statement-based scale. Opinions were further discussed in the focus group. Focus group discussions were transcribed and analysed by a team of investigators. The coding procedures, identifying elements appreciated in group-based principles for action, were thematic and data driven.

**Results:**

Statements about participatory programming generated much less consensus in appreciation among respondents than statements about enjoyment and fostering group processes. To some extent, group members participated in the development of program content. Participation in group formation or community initiatives was less frequently perceived as something within group members’ control. Enjoyment, expressed as physical and emotional experiences, was found to be an individual driver of group exercise. Fostering group processes, expressed as social support, was found to contribute to enjoyment and learning achievements. Responsive leadership, ensuring responsive guidance, by an enthusiastic exercise trainer acting as a role model, were identified as additional necessary principles for action.

**Conclusions:**

Group-based principles for action in CBHEPA programs are not clearly demarcated. Fostering group processes is an overarching principle, conditional for the spin-off in terms of enjoyment and active participation. This, in turn, leads to a sense of ownership among participants, who take up responsibility for the exercise group as well as their individual activity behaviour. CBHEPA programs thrive on participants having fun together and exercise trainers’ leadership skills. A professional, competent, responsive exercise trainer plays a key role in the organisation and maintenance of CBHEPA programs.

**Electronic supplementary material:**

The online version of this article (doi:10.1186/s12889-015-2515-6) contains supplementary material, which is available to authorized users.

## Background

Worldwide, physical inactivity is one of the core risk factors for non-communicable diseases such as diabetes type II and cardiovascular disease [1, 2]. In the Netherlands, sports and physical activity engagement is lower in socially vulnerable groups than in wealthier groups [3, 4]. The Dutch Healthy Physical Activity Guidelines (NNGB) set the norm for healthy daily physical activity for adults at a minimum of daily 30 minutes moderate activity at least five days a week [5]. Socially vulnerable people most at risk of not meeting the NNGB are those of low socio-economic status (SES), or who are unemployed, or of non-Dutch origin, or with chronic disease(s) [4]. To reduce these inequalities in physical activity behaviour, Dutch health policy focuses on the implementation of community-based health enhancing physical activity (CBHEPA) programs [6, 7] in order to improve individual health and wellbeing, to reduce inequalities in health and PA behaviour across population subgroups, and to realise public gains in terms of reduced healthcare expenses [6].

Current theories on enhancing physical activity behaviour and maintenance suggest that physical activity interventions function through individual psychosocial processes (goal-setting, motivation, self-efficacy, and coping with stressors) [8–12], through interactions and group dynamics in exercise groups, and through interactions with the social environment and community [13–19]. Therefore, CBHEPA programs are grounded in individual, group, and community-based theories [20–22].

Dutch CBHEPA programs are built on principles for action for health promotion interventions [7, 23], as advocated by the WHO and others [24, 25]. Since the publication of the Ottawa Charter for Health Promotion [24], professionals are challenged to work explicitly with principles for action important to modern health promotion [25]. A principle describes the code of conduct or a rule of action and is generally action oriented [26]. Principles for action encompass a continuum of values emerging from health promotion research and practice. At one end of the continuum, more conventional health and physical activity promotion principles are found, reflecting traditional health education based on biomedical, behaviourist, and reductionist approaches to health. Usually, these programs address a specific topic or lifestyle, with an emphasis on targeting at-risk people with behaviour change strategies [25]. At the other end of the continuum, health promotion is guided by principles for action based on an ecological perspective on human health [27, 28]. This perspective on health and physical activity promotion emphasises the need for actions that are empowering [29], participatory [30–32], intersectoral, equitable, and sustainable, and that use multiple strategies [33]. The focus is on health as a resource for meaningful living [34–37].

From this latter perspective, it is expected that using principles for action contributes to the effectiveness of CBHEPA programs. Principles for action leave scope for adjustment to contextual needs on the one hand, and are the program’s constituents which can be implemented in different contexts and settings on the other hand [38]. Usually, the effectiveness of CBHEPA programs is based on measuring physical activity outcomes at individual level, using standardised self-report instruments [39], but how defined or ideal principles for action emerge in practice is largely dependent on contextual factors, knowledge, or the skills of the local professionals involved. Whether or not principles for action are recognised and valued by participants in exercise groups in on-going CBHEPA programs, and how they contribute to effectiveness, is rarely investigated.

As part of an on-going evaluation study of a Dutch CBHEPA program, Communities on the Move (CoM) [21], we wanted to explore particularly group-based principles for action, since CBHEPA programs in the Netherlands are generally group-based. CoM was developed and disseminated (2003–2012) by the Netherlands Institute for Sports and Physical Activity (NISB) and targets socially vulnerable groups. CoM defined a set of principles for action at individual, group and program level. This current study aims to evaluate CoM’s group-based principles for action in group settings. It addresses the question which of the identified group-based principles for action are perceived as important by CoM participants. We thereby hope to contribute to the knowledge base on the use and impact of principles for action in group-based physical activity programs, using a practice-based evaluation approach.

## Methods

We studied how participants appreciated the group-based principles for action applied in CoM: active participation, enjoyment, and fostering group processes. An exploratory evaluation design was used. The principles for action were operationalised on the basis of the literature on social cognitive theory [40–42], social learning theory [43], and social capital and participation [30, 31, 44], alongside interviews (*n* = 11) and expert consultation (*n* = 2). **S**cientific [45–49] and grey literature [50, 51] were explored to identify data collection techniques suitable for low literate and culturally diverse socially vulnerable groups. Focus group techniques were identified, alongside cultural sensitive techniques actively engaging the target group, facilitating dialogue and providing immediate feedback. The principles for action were operationalised as follows:

- *Active participation*: 1) participation in group formation [19, 52, 53], 2) participation in physical activity program content decision making [54, 55], and 3) participation in community initiatives [54, 56, 57].
- *Enjoyment of physical activity*: 1) expressions of enjoyment (physical, verbal and nonverbal) [58–60] and 2) safe and supportive environments [27, 52, 61, 62].
- *Fostering group processes*: 1) social support, looking at group composition (size, [cultural] diversity, boundaries, phase) and group structure (roles, norms, social support, and cohesion) [13, 63], 2) role of the exercise trainer [17, 62, 64], and 3) learning achievements [40, 43].

Based on these operationalisations, a semi-structured interview protocol was developed: the active participation, enjoyment, and fostering group processes (APEF) tool, to assess participant appreciation for each of the group-based principles for action (Table 1). For each principle, two or three statements were formulated, allowing data to be collected on individual points of view, as well as probing theme-driven dialogue between researcher and respondents and dialogue among respondents. The development of the APEF tool for group-based principles for action will be described in detail elsewhere (Herens, Wagemakers, Vaandrager, Van Ophem, Koelen, in preparation).

**Table 1:** Outline of the interview protocol (APEF tool)

Principle	Variable	Statement	Examples of in-depth questions
Active participation	Group formation	1. We, as exercise group, choose who participates in the exercise group.	Since when have you been exercising together?
			How are participants recruited?
			Do you ever bring a friend or a neighbour?
	Content activity class	2. We, as exercise group, choose the activities for the exercise class	What does your physical activity program look like?
			Were you involved in the choice of activities, and if so, how did that work?
			How important is that for you?
	Community initiative	3. Some participants within the exercise group take the initiative to exercise together elsewhere	Can you give an example of somebody taking the initiative?
Enjoyment	Enjoyment of physical activity	4. Exercising in the exercise group ensures that I like being physically active	What physical activity do you like most?
			Is the program consistent with your preferences?
			How do you ensure that everybody can enjoy the physical activity class?
	Feelings of safety	5. The exercise group offers me safety to be physically active	What comes to your mind if we talk about safety?
			How does the group support safety?
Fostering group processes	Social support	6. Exercising in the exercise group offers me support to be physically active	What comes to your mind if we talk about group support?
			In what way does the group offer support to physical activity behaviour?
			How do you deal with factors that make physical activity difficult?
	Role exercise trainer	7. Within the exercise group, the exercise trainer is an example for me to be physically active	In what way is the exercise trainer an example?
	Learning achievements	8. By exercising in the exercise group, I learn how to be more physically active in my daily life	Can you give examples of what you learned in the exercise group?
			What have you discovered since you joined the exercise group?
			What is your benefit or achievement?

### Data collection

From May 2013 to May 2014, ten focus groups were conducted in Dutch CBHEPA programs, including exercise groups participating in the CoM evaluation study (convenience sampling). The APEF tool was used in ongoing exercise groups, except for two. In these latter groups, participants still came together as part of an educational scheme (groups 1 and 2). Group members were asked to participate in a focus group. In all ten groups, a number (range 6 to 11) of group members were willing to participate (*n* = 76).

**Table 2:** Characteristics of CBHEPA programs

Focus group	Respondents	Municipality	CBHEPA program					
	*N* = 76			Duration	Sports venue	Frequency	Main activities	Target group
1.	Women^a^ (*n* = 6)	Amsterdam	A	Fixed (10 weeks)	Community centre	Weekly (1.5 h)	Walking/running (Embedded in language class)	Socially vulnerable women (non-Dutch)
2.	Women (*n* = 6)	Enschede	B	Fixed (13 weeks)	Sports club canteen	2 x week (3 h)	Introduction to various sports activities (Embedded in education trajectory, including follow-up meetings once every 6 weeks for 18 months)	Socially vulnerable women (non-Dutch and Dutch)
3.	Women (*n* = 8)	Helmond	C	Continuous	Playground outdoor fitness	Weekly (1 h)	Outdoor group fitness	Socially vulnerable groups (non-Dutch and Dutch)
	Men (*n* = 1)							
4.	Women (*n* = 6)		C	Continuous	Playground outdoor fitness	Weekly (1 h)	Outdoor group fitness	Socially vulnerable groups (non-Dutch and Dutch)
	Men (*n* = 2)							
5.	Women (*n* = 6)	Rotterdam	D	Continuous	Community centre	Weekly (1 h)	Group exercise to music	Socially vulnerable women (non-Dutch)
6.	Women (*n* = 10)		D	Continuous	Community centre	Weekly (1 h)	Group exercise to music, incl. fall prevention	Socially vulnerable women (non-Dutch and Dutch)
7.	Women^b^ (*n* = 11)		D	Continuous	Community centre	Weekly (1 h)	Group exercise to music	Socially vulnerable women (non-Dutch)
8.	Men (*n* = 7)		D	Continuous	Residential care home	Weekly (1 h)	Group fitness class	Socially vulnerable men (non-Dutch)
9.	Women (*n* = 4)	Tilburg	E	Continuous	Community centre	Weekly (1 h)	Group exercise class, incl. fall prevention	Socially vulnerable elderly women and men with a chronic condition (Dutch)
	Men (*n* = 3)							
10.	Women (*n* = 6)		E	Continuous	Community centre	Weekly (1 h)	Group exercise class	Socially vulnerable elderly women, some with a chronic condition (Dutch)

^a^Focus group 1 was conducted during language class in a community centre, in the presence of four migrant women not participating in the physical activity group

^b^In focus group 7, five respondents were not participating in the CoM evaluation study [21]. As a consequence no background details of these respondents were available, except gender and ethnic origin

The focus groups were conducted in rather open settings, using the sports venue (a community centre, sports club canteen, or class room) as meeting place. In four focus groups, outside listeners were present, who were told not contribute to the discussions since they were not participating in the CBHEPA program.

Prior to each focus group, members gave oral consent for their participation and for the proceedings to be audio recorded. The aim and procedure was explained by the researcher (first author). Dutch was the language of conversation in all groups.

Statements were presented during the focus groups, written on flipcharts. Each statement was read out aloud. Respondents were asked to individually score each statement with coloured voting cards carrying both text and symbols: ‘agree’ (green card with ☺); ‘neither agree nor disagree’ (yellow card with ) or ‘disagree’ (red card with ☹). Group scores were reported on the flipcharts during the focus group and further discussed in-depth. The researcher acted as facilitator to generate the free flow of information among respondents. Assistance was provided by one or two junior researchers.

The duration of each focus group ranged from 50 to 70 min. Some women left before the end of one focus group because they had to collect their children or grandchildren from school.

### Ethical considerations

The authors declare that the study was conducted in accordance with general ethical guidelines for behavioural and social research in the Netherlands, stipulating that behavioural research falls outside the scope of the Act on review of medical research involving human subjects (WMO) when a study is not of a medical nature, and subjects do not receive a particular treatment or are asked to behave in a particular way [65]. Furthermore, the study design was peer-reviewed and approved by the review board of the Wageningen School of Social Sciences. All participants entered into the research with voluntary consent. They were provided with information about the purpose and contents of the study. Guarantees of confidentiality and anonymity were given prior to each focus group. Moreover, participants were able to withdraw from the study at any time for any reason.

### Data analysis

Our analytical strategy to identify respondents’ appreciation of group-based principles for action was thematic and data driven [66]. We followed a stepwise procedure [67]: 1) To assess respondents’ individual appreciation, the scores for each statement were counted (one vote, one point) and added up. For final analysis, all scores were added up across the ten groups. 2) All focus group discussions were transcribed ad verbatim. 3) Respondents were de-identified in the transcript. 4) Transcripts were read by at least two researchers. 5) Top-down coding was developed, based on elements identified in the literature, for each group-based principle for action. For example, codes used for a group dialogue on social support were: (group) commitment or engagement, ownership, motivation, task orientation, and collective faith. 6) Coding was extended with codes for ‘responsive leadership’, an additional theme emerging from our data [64, 68]. 7) All transcripts were coded by at least two researchers using Atlas.ti 7.0. Codification differences between researchers were discussed until consensus was reached. 8) For each statement, codes, e.g., size, culture, closed/open groups, were clustered into themes (group composition). Duplicate coding across statements, indicating interrelatedness, was regrouped under one statement. For example, respondents’ views on social support, which were expressed in discussions following the statements both on safety (statement 5) and on social support (statement 6), were regrouped under the statement on social support.

For consistency, the order of statements presented in the results was rearranged compared to the order during interviewing, thus clustering our findings for each principle more concisely. Citations were used to carefully reflect respondents’ language and meanings. Finally, respondents’ views on principles for action in CBHEPA programs were summarised in terms of group-based driving and restraining forces, following Lewin’s group dynamic theory on force fields, to identify what forces matter most in group-based principles for action [69, 70].

## Results

### CBHEPA program characteristics

The content and composition of the ten groups in the CBHEPA programs involved in our study varied (Table 2). Two programs (groups 1 and 2) had a fixed duration (10–13 weeks) and were embedded in educational schemes. Physical activities were intertwined with other (educational) activities in community centres, leading to cross-fertilisation of ideas and activities, e.g., conducting physical activity exercises during language courses. The other eight on-going programs offered exercise classes once or more frequently every week.

In three groups (groups 1, 3, and 4), outdoor activities were organised, such as walking, running, and outdoor fitness in combination with (fall prevention) exercises. In six groups (groups 5 to 10), indoor activities were organised, usually in a community centre, such as endurance training, fall prevention exercises, (folk) dance, aerobics, or zumba. In one group (group 2), a mix of indoor and outdoor activities was organised. The CBHEPA programs predominantly targeted socially vulnerable groups in underprivileged neighbourhoods, e.g., migrant women and men, the unemployed, or elderly people with a chronic condition (Table 2).

### Respondents

A total of 76 respondents participated in the focus groups, 84 % women, 16 % men. Sixty-five percent of them participated for more than six months in the CBHEPA program, whereas others participated for a shorter period (<3 months). Half of the respondents were Dutch, and the other half of non-Dutch origin, representing 15 different countries of origin (e.g., Morocco, Turkey, Syria, Surinam, China, Cape Verde), showing a great ethnic and cultural diversity between and within groups. Household incomes were relatively low, 48.5 % less than €1,350 a month, as also educational levels, with 42.2 % having no, or only primary, education. Additional file 1 summarises respondents’ characteristics.

Exercise groups were rather homogeneous in terms of age. The majority were middle aged, with a mean age of 61.6 years (*sd* 13.2). Groups were also rather homogenous in terms of gender: six groups contained women, one contained men, and three contained men and women. Gender diversity within exercise groups seemed to be linked to homogeneity in origin: participants in the mixed groups were of Dutch origin, usually consisting of (married) couples. Gender homogeneous groups with participants of non-Dutch origin usually represented a heterogeneous mixture of ethnic and cultural origins, challenging both exercise trainers and participants to use Dutch as their common language.

Respondents indicated that group composition varied during each session and over time. Composition and size differed, because ‘*There is always someone not able to come’* due to illness, weather conditions, work, appointments, family obligations, or holidays.

#### Drivers to participate

Respondents’ individual drivers to participate were to (re)gain health, lose weight, meet people and sociability. Respondents often referred to positive physical activity experiences earlier in life in relation to their drivers to participate, some of whom reported up to 60 years of experience. Additional drivers were accessibility and program diversity (educational and social activities). Unsatisfactory experiences elsewhere, such as program or staffing irregularities or lack of variety in activities, were also mentioned as motives to participate in the current CBHEPA programs.

### Respondents’ appreciation of group-based principles for action

**Fig. 1:**
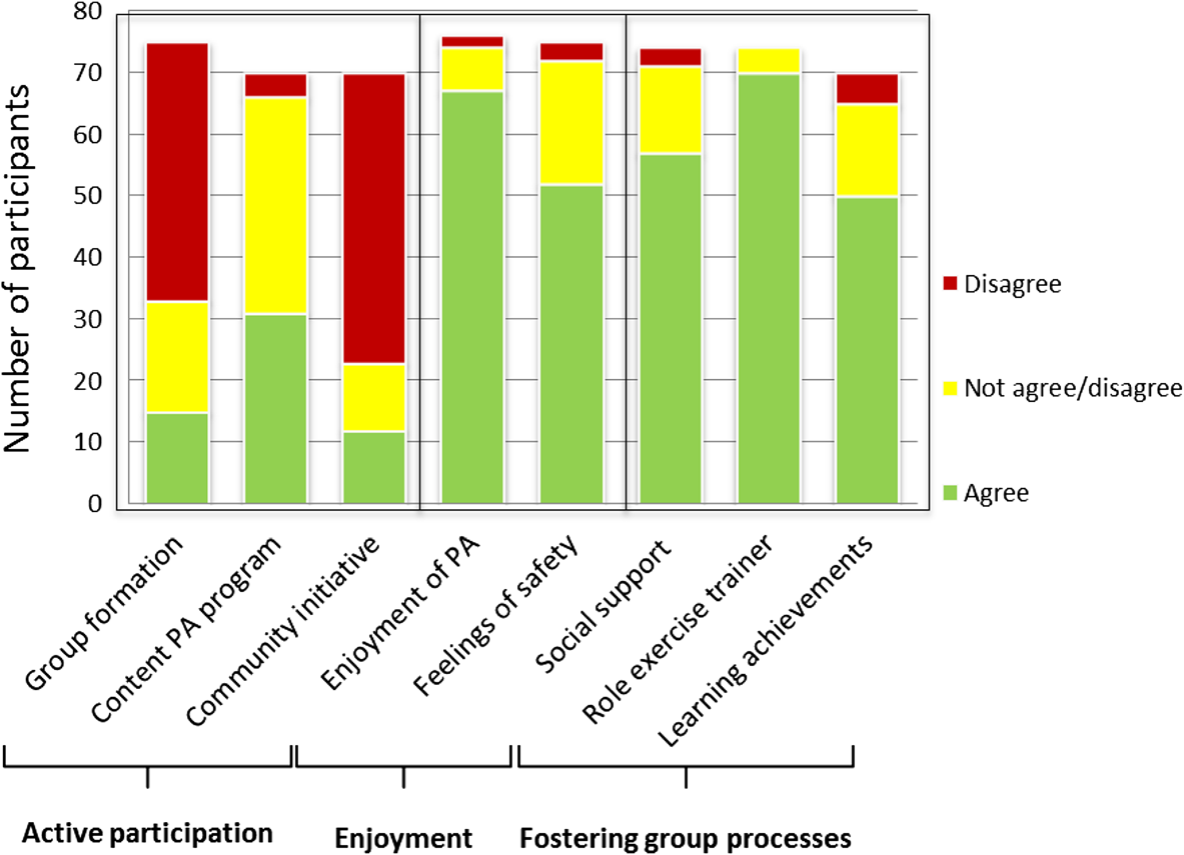
Overall scores per statement for group-based principles for action (*n* = 76)

Overall scores on the eight statements across the ten focus groups show that statements about active participation generated much less agreement among respondents than statements about enjoyment. The greatest consensus was reached for statements about fostering group processes, in particular regarding the role of the exercise trainer (Fig. 1).

#### Active participation as a principle

##### Participation in group formation

Statement 1: ‘*We, as exercise group, choose who participates in the exercise group’* was scored by 75 respondents. Over half of them, 56% (*n* = 42), disagreed, and 24% (*n* = 18) neither agreed nor disagreed. Most respondents were of the opinion that they did not choose who participated in the exercise group, nor were they in control of group formation, since ‘*everyone decides for him/herself*’. Some indicated that, particularly at the start of a program, the exercise trainer played a crucial role in recruiting participants. Exercise trainers took care of publicity (leaflets, face book, newspaper) and word-of-mouth advertising, or mobilisation of local key persons to advocate the program, for example in a community centre, church, or mosque.

*R: She first started in the mosque, the Turkish mosque. That’s how I heard about it, from the people who were going to the mosque. We’d go to the mosque first, we’d exercise there.* <FG7>

Other methods of group formation were referral by a general practitioner, social worker, or work coach as part of – sometimes obligatory – social activation schemes.

In long-standing exercise groups, respondents indicated that there was a regular influx of new participants. Open boundaries and willingness to accept differences were mentioned as relevant factors for the maintenance of exercise groups. Group members’ participation in group formation increased when group maintenance became a shared interest of members and the exercise trainer. A combination of strategies, in which both exercise trainer and group members recruited new people, was then used. Respondents mobilised their social networks, using personal beneficial experiences as motivating messages.

*R: And the strange thing is, when someone new joins, there’s this “click”. None of us has any problem with it at all.* <FG3>

Sometimes, new participants as well as irregular attendance were mentioned as causes for dissatisfaction within the group because of differences in physical activity skills between beginners and advanced participants.

##### Participation in content development of the CBHEPA program

Statement 2: *‘We, as exercise group, choose the activities for the exercise class’* was scored by 70 respondents. Forty-four percent (*n* = 31) agreed with this statement, and 50% (*n* = 35) neither agreed nor disagreed. Most respondents held the opinion that they did not choose the program activities, although opinions also differed. Some felt free to make suggestions about the physical activity program, whereas others felt it was generally the exercise trainer who planned and decided upon program activities. Respondents attributed their program satisfaction to the exercise trainer and his/her sensitivity to participants’ needs.

*R: Well, maybe we have something to say about it, but we just leave that job to the exercise trainer.* <FG10>

Participation in the content of the physical activity program was linked to everyone’s individual responsibility for healthy exercising, their own awareness of (physical) limitations, and their ability to communicate this to the exercise trainer.

##### Community initiative and sport participation

Statement 3: *‘Some participants within the exercise group take the initiative to exercise together elsewhere’* was scored by 70 respondents. Sixty-seven percent (*n* = 47) disagreed. Participation in community initiatives or exercising together elsewhere, in addition to the CBHEPA program, was not perceived as a result of the exercise group. Some respondents reported additional sports participation, e.g., a fitness club, mostly in groups where CBHEPA program activities had stopped. This was perceived as a result of individual rather than group-based actions. Others – mostly respondents of Dutch origin – indicated that they were habitually engaged in leisure-time sport (e.g., swimming, badminton), in addition to the CBHEPA program. As they explained, they were ‘*used to doing sport in leisure time since childhood*’.

Respondents indicated that they occasionally became involved in organising a community initiative, such as physical activity events or other kinds of activities (shopping, city trips). The exercise trainer often acted as an initiator.

*R: Some of us go in that 24-hour charity run against cancer. The exercise trainer puts the idea on the table and says this or that about it. Then some of us take it up and talk about it a bit more. That’s how it goes.* <FG3>

#### Enjoyment as a principle

##### Enjoyment experienced in physical activity

Statement 4: *‘Exercising in the exercise group ensures that I like being physically active’* was scored by 76 respondents. The majority, 88 % (*n* = 67), agreed. Enjoyment was unanimously perceived as a result of the exercise group. Respondents mentioned mostly examples of physical and nonverbal experiences of enjoyment, such as ‘feel the energy’, ‘feel your body move’, laughter, sense of freedom, but also enjoying relaxation after physical exertion, e.g., while taking a shower. Respondents indicated that enjoyment was closely related to program satisfaction, e.g., the nature of activities and the ease with which they could incorporate physical activity in their daily routine. In addition, the exercise group offered an environment for self-expression and escape from daily duties, thereby contributing to enjoyment.

*R: Well, the dancing just makes you happy. Because the energy inside you gets out, so all the emotion comes out too.* <FG2>

##### Feelings of safety

Statement 5: *‘The exercise group offers me safety to be physical active’* was scored by 75 respondents. Sixty-nine percent (*n* = 52) agreed, and 27 % neither agreed nor disagreed. The safety offered by the exercise group was not unanimously perceived as a result of group activities. Discussions about the statement revealed that some respondents defined safety as *environmental* safety, highlighting security of materials, sports venues, and protection against loss or theft. Others defined safety as *emotional* safety, highlighting mutual care and respect, e.g., no prying eyes, dress codes, or being ridiculed or criticised.

*R: I had a different kind of safety in mind < … > I thought to myself, here I am dancing with my fat ass and I’m doing everything wrong and I just don’t care. That was what I was thinking.* <FG4>

Feelings of safety during the exercise class seemed a prerequisite for enjoyment, contributing to individual program adherence, group cohesion, and group maintenance. Group members encouraged feelings of safety by being sensitive, refraining from judgements, and looking out for one another’s (physical) safety.

#### Fostering group processes as a principle

##### Social support in the exercise group

Statement 6: *‘Exercising in the exercise group offers me support to be physically active’* was scored by 74 respondents. Seventy-seven percent (*n* = 57) agreed, and 19 % neither agreed nor disagreed. The social support offered by the exercise group was unanimously perceived as a result of group activities. Social support contributed to enjoyment and feelings of safety during exercise class. Forming partnerships was given as an example: duos of participants helping each other throughout the exercise class. Complimenting and helping one another, and enthusiasm, strengthened respondents’ program adherence and physical activity maintenance.

*R: And sure, the enthusiasm of the group and every time it’s like “oh!” then you get another compliment < … > At a certain moment it gives you wings and then. Now I’m beginning to like this [physical activity].* <FG4>

Social support appeared to go beyond the exercise group in reaching out to non-attending group members (making inquiries, telephone calls, home visits). Respondents indicated that they were closely involved in one another’s lives. In some exercise groups, a group leader was assigned to this particular role, assisting the exercise trainer in organising and motivating fellow group members. In other groups, group roles were less personalised and varied over time in relation to the goal or task achievement of the exercise group. Examples of group roles encouraging social support were: the achiever, the initiator, the joker (fun), and the helper.

The social support offered by the exercise group was enforced by the shared group norm that physical activity is healthy and fun to do.

*R: I just like it, for my health. Physical activity is good for you, everyone knows that.* <FG7>

Other enforcing group norms were acceptance of diversity (e.g., in culture, opinions, health status, literacy rate, or physical activity skills), encouraging one another during and outside the physical activity classes, and sharing knowledge about a healthy and active lifestyle. Respondents of non-Dutch origin (both men and women) specified tolerance of dress codes and a need for secure sport environments. Social support was also enforced by organised time and opportunity for socialising as part of group activities.

##### The role of the exercise trainer

Statement 7: *‘Within the exercise group, the exercise trainer is an example for me to be physically active*’ was scored by 74 respondents. Ninety-five percent (*n* = 70) agreed. The exercise trainer was perceived as a role model to be physically active by nearly all respondents, in terms of personality (being open and kind) and physical appearance (being slender, fast, agile). Respondents expressed great confidence in their exercise trainer to guide and support them during the exercise classes. They were of the view that a professionally trained exercise trainer contributed to confidence building, and that a well-organised exercise trainer, taking care of planning, time management, group continuity, and maintenance, also contributed to personal confidence and belief in task performance. Respondents trusted the exercise trainer in selecting activities tailored to their needs.

Alongside professionalism, a positive disposition (e.g., optimism, cheerfulness, witty, putting things in perspective) was mentioned as a key quality of an exercise trainer, as well as the willingness to share personal experiences (e.g., dealing with pain or discomfort while exercising).

*R: He [exercise trainer] is always cheerful, always optimistic. He presents it really well, with jokes and all that. He’s just great.* <FG4>

Relationship development was fuelled by the exercise trainer’s responsive guidance: attentiveness to program adherence and sensitivity to each participant’s individual conditions.

*R: The exercise trainer watches to see whether you are doing it right for your own body or not. He knows about my pain complaints and he’ll tell you; you’re doing it wrong, you have to do it like this.* <FG3>

In long-standing exercise groups, bonding between exercise trainer and group members was reported. The exercise trainer was considered a friend as well as an expert. Examples were given of how respondents followed their trainer in different activities at various locations. Other examples illustrated how classes failed as soon as the exercise trainer was absent. Attendance rates dropped or activities were not conducted, despite the fact that group members knew their exercises quite well.

*R: If we have to do it ourselves, we don’t get very far < … > We try to start by ourselves, but it lasts for about three counts, and then it just blocks < laughs>.* <FG10>

##### Learning achievements

Statement 8: *‘By exercising in the exercise group, I learn how to be more physically active in my daily life’* was scored by 70 respondents. Seventy-one percent (*n* = 50) agreed, and 21% (*n* = 15) neither agreed nor disagreed. Most respondents perceived physical activity learning achievements as a result of exercise group activities. Respondents who agreed referred to personal learning achievements relating to perceived benefits, awareness, and the ability to integrate physical activity in daily life. Respondents differentiated between perceived direct benefits and long-term returns. Direct benefits were mostly experienced wellbeing, feeling more energetic and fitter, and sense of accomplishment. Long-term returns were mostly better posture and limberness, keeping balance, and weight loss.

Respondents mentioned increased organisational ability to integrate physical activity into their daily life. For some, weekly participation in the CBHEPA program was helpful in planning and structuring their physical activity behaviour. Practical instructions about how to practice exercises in daily life helped to increase both awareness and actions outside the lessons. All agreed that self-management and self-organisation, by scheduling physical activity in daily activities, e.g., exercises at home, while cooking, washing the dishes, or walking the dog, were most important for physical activity maintenance. Respondents mentioned increased physical abilities through observational learning, imitating the exercise trainer’s movements. They were also role models for one another when trying to keep up with the exercise, or when not catching instructions (e.g., as a result of deafness).

*R: You imitate a thing or two. The exercise trainer joins in too [in the exercises].* <FG3>

Respondents repeatedly mentioned regaining physical abilities, lost due to chronic illness or aging. Concrete examples were: learning to walk without a stick, moving around without a rollator, riding a bicycle, regaining balance. As a result, respondents indicated that they felt more confident, self-reliant, and better able to manage physical activity in daily life, thereby contributing to their wellbeing.

### Driving and restraining forces for group-based principles for action

During the focus groups, respondents mentioned various positive and negative aspects of group-based principles for action, thereby defining the driving and restraining forces relating to the processes and group dynamics in their exercise groups. Summarising these views revealed an interplay between the efforts put into the process of group development on the one hand, and group members’ efforts put into personal goal attainment on the other. Respondents indicated that they started the program for personal, usually health-related, reasons or as a meaningful leisure-time activity. Initially driven by individual needs and goals for physical activity behaviour, respondents shared experiences about their development as group members, taking responsibility for group atmosphere, task achievement, and group maintenance. The longer the group was in existence, the more the participants’ boundaries opened up within the (safe) context of the group, enabling enjoyment, experiential learning, and group development. Also, the personal boundaries of the exercise trainer opened up, and hence he/she became a friend as well as an expert.

Key drivers at individual level in this process can be summarised as self-awareness and sense of interdependency. Key drivers at group level can be summarised as social support (among group members) and responsive leadership, mostly acted out by all parties as communicative skills. Restraining forces can be summarised as too many or hard-to-manage differences within a group, e.g., in performance (physical activity skills and aims), in age, or in personalities, and lack of time or opportunity to organise dialogue (Table 3).

**Table 3:** Driving and restraining forces for principles for action in exercise groups

Principle for action	Driving forces (+)	Restraining forces (−)
*Active participation*		
Group formation	▪ using personal beneficial experiences as motivating messages in social network	▪ irregular attendance
	▪ tolerance of newcomers, open group boundaries	▪ too much difference in physical activity skills between beginners and advanced participants
	▪ exercise trainers seeking publicity and mobilising key persons	
	▪ acceptance of group maintenance as a mutual group interest	▪ lack of ownership and/or interest in group maintenance
Content activity class	▪ taking responsibility for oneself in communication and action	▪ lack of time/opportunity for dialogue
	▪ awareness of personal limitations in practicing physical activity	▪ poor language skills or health literacy
	▪ exercise trainer’s sensitivity to personal needs	▪ change in exercise trainer
Community initiative	▪ exercise trainer acting as initiator	▪ lack of participants’ interest or support
	▪ shared responsibility for group activity	▪ lack of (additional) leisure time
*Enjoyment*		
Enjoyment	▪ exercises aimed at cooperation and nonverbal communication	▪ lack of physical activity skills
	▪ providing for energising experiences, relaxation exercises, and playfulness	▪ lack of variety in activities
	▪ interpersonal attractiveness	▪ lack of sensitivity to individual needs
	▪ use of music of participants’ past or country of origin	▪ lack of sensitivity to individual backgrounds
Feelings of safety	▪ minding one another’s (physical) safety	▪ lack of sensitivity to one another
	▪ secure physical activity environments	▪ lack of (perceived) safety of physical activity material or sports venue
	▪ being sensitive to one another	▪ judging one another
	▪ helping one another, giving assistance	
	▪ trust and mutual respect	▪ lack of mutual trust and respect
*Fostering group processes*		
Social support	▪ care for non-attenders (reaching out, visit)	▪ lack of time or opportunity to socialise during exercise class
	▪ encouraging one another during and outside the physical activity classes	
	▪ sharing knowledge about a healthy and active lifestyle	
	▪ making group roles explicit during classes (group leader, helper, partner)	
	▪ shared norms about group behaviour, e.g., timeliness	
	▪ shared norms about physical activity (healthy)	
	▪ tolerance of dress codes	▪ lack of tolerance
	▪ acceptance of diversity (e.g., in culture, opinions, health status, literacy rate, or physical activity skills)	
Learning achievements	▪ in culturally diverse groups, use of Dutch as common language	▪ use of native language among one another
	▪ practical instructions about how to practice exercises in daily life	▪ lack of group support
		▪ too much involvement in day-to-day concerns
	▪ learning by imitating exercise trainer or fellow participant	▪ too much difference between trainer and participants (e.g., age or phase of life, attitude, outfit)
Role exercise trainer	▪ organising time and opportunity for socialising	▪ program or staffing irregularities
	▪ sensitivity to individual needs, selecting activities tailored to personal needs	▪ lack of target group involvement
	▪ well-prepared (good planning and time management)	
	▪ managing differences and group maintenance	▪ lack of sensitivity to group dynamics
	▪ taking the initiative	▪ lack of physical activity knowledge or initiative
	▪ professionally trained in physical activity and healthy lifestyle	
	▪ acting as a personal coach	
	▪ enthusiasm, positive disposition	
	▪ willingness to share personal experiences	▪ frequent staff changes
	▪ responsive guidance	▪ too much difference/distance between trainer and participants (e.g., age, phase of life, attitude)
	▪ being a friend as well as an expert	▪ expert instead of egalitarian perspective

## Discussion

Our study on respondents’ appreciation of group-based principles for action in Dutch CBHEPA programs – active participation, enjoyment, and fostering group processes – revealed some interesting new insights. Relating to the principle of *active participation,* our findings indicate that group members’ active participation in group formation occurs only after they have participated for some time and happens primarily through sharing beneficial experiences in personal social networks. Initial group member recruitment is perceived as a task for the exercise trainer, through seeking publicity and mobilising key persons.

According to respondents, active participation in the development of content for the CBHEPA program is mostly directed at tailoring activities to individual needs. Tailored programming is highly appreciated; this is in line with other studies [52, 55], endorsing its importance for on-going engagement of socially vulnerable groups in physical activity programs. In addition, our findings make explicit that tailored programming happens provided the exercise trainer knows the sort of participants with whom he/she is dealing and takes the initiative to act on that. This emphasis on the need for exercise trainers to be responsive in physical activity programs has also been found in other studies [62, 64].

Dutch CBHEPA programs aim to empower socially vulnerable groups by improving participants’ health and wellbeing through physical activity. They are developed on the assumption that socially vulnerable groups will become more self-reliant in organising their physical activity behaviour and participate more often in community initiatives. According to our findings, joining a CBHEPA program is respondents’ distinct way of becoming engaged in community initiatives. Only a few of them are engaged in additional sports or community-related activities. One explanation might be that people take part in a CBHEPA program primarily for individual satisfaction, e.g., enjoyment and relaxation, without a desire to pursue collective goals [71, 72]. Another explanation might be that, in practice, Dutch CBHEPA programs use rather conventional health education principles for action, targeting at-risk groups and using a behaviourist and reductionist approach to health, rather than health promotion principles for action, based on an ecological perspective on health [25, 27, 28].

Relating to the principle of *enjoyment of physical activity*, our findings indicate that having fun together is perceived as an important principle for action for program adherence in socially vulnerable groups. The relationship between leisure-time activity and health is a growing area of research, with a particular focus on affective responses, mood and emotions. Experiencing positive affective states through leisure-time (physical) activities is one of the important factors that maintain and promote individuals’ psychological, social, and physical health and wellbeing, by direct strengthening of their health and wellbeing, and as a means of moderating stress or stress effects [73]. In physical activity interventions, enjoyment is found to be a moderator of efficacy [74]. Studies indicate that not only self-control and discipline, but also enjoyment, pleasure and ‘not worrying’, are key values in maintaining an active and healthy lifestyle [58, 75, 76]. In discussing enjoyment, respondents mentioned predominantly individual experiences, described by Jallinoja et al. as ‘negotiated pleasure’, referring to the process of balancing between health-seeking and pleasure-seeking behaviour. Because of a potential discrepancy between these two aims, pleasure is constructed not simply as a spontaneous experience, but often as a planned and disciplined event [46]. ‘Negotiated pleasure’ regarding physical activity, as found in our study, evolves around: 1) pushing oneself, or using someone else as an external push, to overcome the temptations of remaining inactive; 2) the instrumental values of physical activity, such as health or psychological benefits; 3) the satisfaction of physical activity goal achievement; and 4) the physical sensation that is felt during and after being active [46].

Our findings relating to group experiences of enjoyment, expressed as feelings of safety, safe environments, and social support, show that (changes in) affective responses at individual level are strongly linked to group-based experiences, which can be facilitated [77]. This is consistent with the self-determination theory, indicating that, alongside perceived autonomy and competence, relatedness (with fellow participants as well as with the exercise trainer) is an important medium for change and internalisation of physical activity behaviour [8, 9, 78].

Our findings relating to *fostering group processes* illustrate the importance of group support. In discussions on the statements on safety and social support, very similar views emerged, showing an interrelatedness of (emotional) safety and social support. This highlights the important role of interpersonal factors in group-based CBHEPA programs, such as mutual trust, interdependency, respect, attractiveness, integration and sense of belonging. Our findings are supported by other studies on group dynamics in physical activity programs [13, 19, 79]. Group dynamics in CBHEPA programs are, however, often implicit and left unaccounted for. CBHEPA programs are usually group-based for organisational reasons (cost-covering), rather than for behavioural change reasons. Nevertheless, some studies indicate that group dynamics strategies, explicitly applied in group-based physical activity interventions, are more effective in establishing physical activity behaviour change than individually targeted interventions with social support, which, in turn, are more effective than individual interventions without additional social support [16, 22]. At the same time, a lack of standardisation across the literature in relation to how group dynamics strategies are applied in physical activity programs is also reported [16, 18].

Our findings indicate that an exercise trainer acts as a role model in being fit and healthy, as well as in being kind and responsive. Respondents attribute great value to the fact that the exercise trainer is an expert as well as a friend, facilitating learning processes in various domains. Exercise trainers use the exercise group as a relatively convenient environment to bridge (cultural) diversity, using exercises to enhance both verbal and nonverbal communication and cooperation.

Responsive leadership thus emerges as an additional principle for action in group-based CBHEPA programs. Alongside the role model aspect, exercise trainers’ responsive leadership skills are emphasised by respondents. Our study illustrates the need for ‘enabling’ professionals in exercise groups targeting socially vulnerable people [80]. Based on the literature, three areas of expertise can be defined for responsive leadership to facilitate learning processes for behavioural outcomes in such groups: first, the responsibility to ensure that the demands of the organisation are satisfied (satisfactory group size, cost-covering level), and that group members’ needs and aspirations are satisfied [17, 64]; second, the leadership skills to manage resources (ensuring secure physical activity environments, monitoring adherence, fostering group processes), personal reputation and image (being a qualified and enthusiastic role model), and development of relationships (based on [cultural] knowledge, prior experiences, and responsiveness to participants’ performance styles) [68]; third, teaching skills to adapt exercise classes to participants’ knowledge, skills, and (cultural) dispositions: this is probably best described as ‘culturally responsive teaching’ [81].

There is need to further explore the reciprocal relationship between experiential learning within groups (who learns what, when, and from whom), the development of group norms, group cohesion, skills and collective efficacy, and individual behavioural outcomes, such as increased physical activity behaviour and maintenance [16, 82]. This calls for a more systematic approach to determine underlying causal mechanisms of group-based CBHEPA programs [83, 84], to determine how to measure important variables consistently, such as group environment in terms of process and structure, and to compare and contrast across studies [16].

Our study reveals that the group-based principles for action, as defined in CoM, are not demarcated entities, but rather represent a range of intertwined values and principles to organise (group) processes [25, 37]. Fostering group processes seems an overarching principle, conditional for the spin-off in terms of enjoyment and active participation, which, in turn, leads to (the development of) perceived sense of ownership and to participants taking responsibility for the exercise group’s as well as their own physical activity behaviour. Scientific literature on the use and appreciation of group-based principles for action in CBHEPA programs seems fairly limited [25, 33]. Also, in practice, the use of group-based principles for action is rarely made explicit within and across CBHEPA programs, seemingly driven by tacit knowledge and common sense [13, 79]. With our study, using a practice-based evaluation approach, we aim to contribute to the knowledge base on the use of group-based principles for action in CBHEPA activity programs. Our study thus contributes to the on-going discourse on how to improve health-enhancing physical activity interventions [39, 83].

Implications for future research are that proxy indicators or indirect measures need to be identified to assess transformative changes within the group or community [85, 86], and that responsive evaluation strategies should be used, e.g., two-way methods (including group discussions and face-to-face engagement) in order to pick up differing kinds of views, including the use of peer-led questioning [87]. The strength of our study is that we have developed a systematic way of assessing participant appreciation of group-based principles for action. This adds to existing methods of measurement, e.g., individual questionnaires, which are most commonly used to assess outcomes of group dynamics in exercise groups [18, 88, 89].

### Methodological considerations

Some comments on this research relate to data collection and processing. Focus groups varied in composition and size. In some groups, all members were of Dutch origin; in others, a large ethnic and cultural diversity was found. The fact that it was necessary to use Dutch as the common language hindered some respondents from expressing themselves freely in their mother tongue, but challenged others to practice their skills in the Dutch language. Occasionally, those who spoke Dutch fluently translated for others. Therefore, we cannot rule out the possibility that socially desirable responses entered our data set, also because the focus groups were held in existing group settings.

Furthermore, literature on culturally appropriate health and physical promotion offers several strategies to address socio-cultural differences within and between groups [90], such as soliciting input from population members, linking intervention content with values, addressing language and literacy challenges, incorporating population media figures, using culturally relevant forms of physical activity, and addressing specific population linked barriers to activity [91]. Our findings reflect examples of these strategies being used, except the use of media figures. Nevertheless, we cannot rule out possible influences of different beliefs about health concepts across cultures, lack of health literacy or skills in reading, leading to differences in understanding and interpreting the statements [92, 93], despite our positive experience of getting respondents engaged in a meaningful dialogue about group-based principles for action in CBHEPA programs in all focus groups.

The APEF tool, based on statements and subsequent group discussions, proved useful for engaging respondents in a meaningful dialogue. On the positive side, it allowed all respondents to participate. It enabled the researcher/facilitator to reach out to those who kept silent. It also kept respondents alert throughout the focus group. The voting procedure itself was, however, sometimes hard to manage as respondents started discussing as soon as they heard the statement, without using their vote cards and casting their votes only after discussion. Two statements, those addressing social support and group safety, generated considerable debate. It might be that the concepts were too generic and abstract for this target group. In future, safety should be addressed more explicitly in two statements: one addressing environmental safety and the other addressing emotional safety.

Our findings are based on a volume of ten focus groups, including 76 respondents, generating a fairly solid basis for interpretation of our data. The APEF tool also generated data for comparison between groups; this is an indication of its generalisability (external validity).

## Conclusions

In the participants’ eyes, group-based principles for action in CBHEPA programs are not clearly demarcated. Fostering group processes is an overarching principle, generating feelings of safety and social support, which are conditional for the spin-off in terms of physical activity enjoyment and active participation. This, in turn, leads to (the development of) perceived sense of ownership, with participants taking responsibility for the exercise group as well as their own physical activity behaviour. Participants identified responsive leadership as the most important principle for action. A professional, competent, responsive exercise trainer plays a key role in the organisation and maintenance of CBHEPA programs.

## Additional file

Additional file 1:
**Descriptives of focus group respondents.** (DOCX 15 kb)

## Abbreviations

CBHEPA: Community-Based Health Enhancing Physical Activity
CoM: Communities on the Move
NISB: Netherlands Institute for Sport and Physical Activity
WMO: Act on review of medical research involving human subjects
PA: Physical activity
