# Supplementary material for: Exploring participant appreciation of group-based principles for action in community-based physical activity programs for socially vulnerable groups in the Netherlands
Source: BMC Public Health. 2015 Nov 25;15:1173. doi: 10.1186/s12889-015-2515-6 (PMC4659218; doi:10.1186/s12889-015-2515-6)
Supplement: Additional file 1: — Descriptives of focus group respondents. (DOCX 15 kb) [file 12889_2015_2515_MOESM1_ESM.docx]

**Additional file**

**Table S1.** Descriptives of focus group respondents

| **Variable** | **Value** |
| --- | --- |
| ***Socio economic conditions*** |  |
| Gender (n=76)  Women  Men | %  84.0  16.0 |
| Age (n=71)  Mean *(sd)* | 61.6 *(13.24)* |
| Income (n=66)  < € 1,000  €1,001 – €1,350  €1,351 – €1,800  > €1,800  Income not specified | %  22.7  25.8  16.7  10.6  24.2 |
| Education (n=71)  No/Primary education  Secondary education  College/University education  Other | %  42.2  46.5  9.8  1.5 |
| Living conditions (n=71)  Single household  2-person household  > 2-person household | %  35.8  29.9  34.3 |
| Working conditions (n=71)  Work (full-/part-time)  Unfit for work/Retired  Unemployed/Social benefit  Housekeeper | %  8.5  33.8  15.5  57.7 |
| Ethnic origin (n=76)  Dutch  Non-Dutch** | %  50.7  49.3 |
| (If of non-Dutch origin) Years in the Netherlands (n=35)  Mean *(sd)* | 27.3 *(12.55)* |
| ***Quality of Life*** |  |
| Overall score Health-related Quality of Life*  Mean *(sd)* | 6.5 *(1.47)* |
| EQ VAS (0–100)*  Mean *(sd)* | 74.2 *(15.25)* |
| Life satisfaction (0–10)  Mean *(sd)* | 8.0 *(1.47)* |
| Sense of coherence (SoC3) (n=71)  Strong SoC  Moderate SoC  Weak SoC | %  18.5  58.5  23.0 |
| BMI (n=65)  Mean *(sd)* | 29.3 *(4.85)* |
| ***Physical Activity*** |  |
| Participation duration in PA program (n= 69)  < 3 months  3–6 months  > 6 months | %  26.1  8.7  65.2 |
| Member sports club (n=63)  Yes  No | %  20.6  79.4 |
| Total PA minutes/day  Mean *(sd)* | 210.1 *(147.15)* |

* Health-related QoL was measured with EuroQoL 5D-3L; overall score 5 = no problems/complaints; score 15 = severe problems/complaints; EQ-VAS (EuroQoL visual analogue scale scores today’s self-perceived health, scale 0–100).
** Among n=76, 15 different countries of origin were identified.
